# Acceptability and Feasibility of Human Papillomavirus Vaccine Introduction in Cameroon: A Mixed-Methods Study

**DOI:** 10.7759/cureus.60723

**Published:** 2024-05-20

**Authors:** Etienne Guenou, Blaise Wakam Nkontchou, Marius Vouking Zambou, Collins Buh Nkum, Annick Collins Mfoulou Minso, Yves Legrand Napa, Landry Beyala Bita'a, Augustin Murhabazi Bashombwa, Ketina Hirma Tchio-Nighie, Jerome Ateudjieu, Eugénie d'Alessandro

**Affiliations:** 1 Epidemiological Surveillance Section, National Public Health Laboratory, Ministry of Public Health, Yaoundé, CMR; 2 Department of Health Research, Meilleur Accès aux Soins de Santé (M.A. SANTE), Yaoundé, CMR; 3 Faculty of Medicine, Aix-Marseille University, Marseille, FRA; 4 Global Polio Eradication Initiative, Ministry of Public Health, Yaoundé, CMR; 5 Supply and Logistic Unit, United Nations International Children's Emergency Fund (UNICEF) Country Office, Yaoundé, CMR; 6 Department of Public Health, Faculty of Medicine and Biomedical Sciences, University of Yaoundé, Yaoundé, CMR; 7 Department of Public Health, University of Dschang, Dschang, CMR; 8 Division of Health Operations Research, Ministry of Public Health, Yaoundé, CMR; 9 Municipal Hygiene and Health Service, Ville de Salon-de-Provence, Salon-de-Provence, FRA; 10 Centre Norbert Elias, École des Hautes Études en Sciences Sociales (EHESS), Marseille, FRA

**Keywords:** epi, cameroon, cervical cancer, perceptions, hpv vaccine, human papillomavirus

## Abstract

Introduction: Human papillomaviruses (HPV) are responsible for sexually transmitted infections, and some of these viruses have oncogenic potential. The HPV vaccine is due to be introduced in Cameroon in September 2019. Our study looked at the knowledge, perceptions, and attitudes of the population and healthcare professionals regarding cervical cancer and its vaccine prevention. This approach provides a solid basis for, among other things, developing a clear communication strategy for the introduction of the vaccine.

Objective: This study aimed to assess the feasibility and acceptability of introducing the HPV vaccine in Cameroon among key stakeholders including health workers and parents.

Methods: From March to May 2019, we conducted a qualitative and quantitative descriptive study in six health districts in the Centre Region. A total of 257 study participants were recruited, including 168 parents and 89 health professionals; 60 interviews were also conducted, 30 with parents and 30 with health professionals. The quantitative data collected were analyzed using IBM SPSS Statistics for Windows, Version 20.0 (Released 2011; IBM Corp., Armonk, New York, United States); for the qualitative analysis, we carried out repeated readings of the transcribed interviews. This work enabled us to identify the significant themes emerging from the interviewees' discourse.

Results: The vast majority of healthcare professionals claim to be aware of cervical cancer (93.3%), but only 15.7% of female healthcare professionals claim to have ever carried out a screening test. A significant proportion of these professionals have actual experience of cervical cancer. Among parents, knowledge of this cancer also appears to be relatively high for a lay audience (54.2%), with a low screening rate (7.1%). Awareness of the HPV vaccine as a cervical cancer prevention tool was very low: 14.9% among parents and 44.9% among healthcare professionals. In addition, we found that information about the existence of an HPV vaccine was still very low among parents (83.9% had never heard of it); 43.8% of healthcare professionals had been informed about the vaccine at their training school. As regards acceptance of the HPV vaccine, the quantitative and qualitative results point in the same direction. The majority of parents are in favor of a campaign and access to this new vaccine via the Expanded Program on Immunization (EPI). However, many of them (94.6%) explained that they wanted more information before making a decision.

Conclusion: Informing and raising public awareness of cervical cancer, the HPV vaccine, and vaccine safety are essential measures to encourage public support for the HPV vaccination campaign.

## Introduction

Human papillomaviruses (HPV) are responsible for sexually transmitted infections [1]. Some of these viruses are cancer-causing, in particular strains 16 and 18, which can cause cancer of the cervix, vulva, anus, penis, and throat. The non-oncogenic strains (6 and 11) also have health repercussions, such as anogenital warts (anus, perineum, penis, vulva) [1,2]. Cervical cancer is the leading cause of cancer deaths (about 80%) among adult women in developing countries and the second most common cancer in women worldwide, with nearly half a million women diagnosed each year [1]. In Cameroon, there are more than 2,000 cases, with a proportion of around 30 new cases of cervical cancer per 100,000 women per year [3]. This level of prevalence places Cameroon among the countries with the highest prevalence of cervical cancer in the world. Most Cameroonian women do not have access to effective screening and early treatment programs that could halt or slow the progression of the disease. New vaccines designed to prevent infection by the HPV, the main cause of cervical cancer, have the potential to protect new generations of girls from this cancer. A great deal of research points in the same direction [4,5] and shows that the quadrivalent vaccine for HPV (6, 11, 16, and 18) is considered to be one of the strategies for reducing cervical cancer [6], with 80-100% protection of vaccinated subjects against anogenital warts and 60-80% against precancerous lesions.

In Cameroon, in September 2019, the quadrivalent vaccine for HPV (6, 11, 16, and 18) will be adopted in the Expanded Program on Immunization (EPI), targeting adolescent girls aged 9-13 [7]. The implementation process and adequate vaccination coverage will depend on public support for vaccination, knowledge of HPV and its impact on health, and an integrated approach among adolescents, parents, and healthcare professionals [8,9]. In general, studies have shown that in the past and currently, communities have had, and still have, mixed reactions to vaccination [10,11]. These reactions have been described as being due to a variety of factors: from the political situation linked to the administrative authorities who carried out the vaccinations to the perception that vaccination conflicted with local traditions and to what were perceived as intrinsic manufacturing defects [10].

As part of the HPV vaccination campaign project in Cameroon, our study is proposed to assess the knowledge, perceptions, and attitudes of the parents and health professionals concerning cervical cancer and its prevention by vaccination on the one hand and to assess the feasibility and acceptability of introducing the HPV vaccine in Cameroon among key stakeholders including health workers and parents on the other hand. Furthermore, this study will help to inform future vaccination strategies given that our work falls within the framework proposed by the WHO for the introduction of new vaccines. The UN agency states that this type of research is an essential step in the planning of a vaccination program. This approach provides a solid basis for developing a clear communication strategy, among other things [12]. 

## Materials and methods

Design

This is a qualitative and quantitative descriptive study. The study took place from March to May 2019. Participants were recruited from six health districts in the Centre Region, including three urban districts (Biyem-Assi, Cité Verte, Efoulan) and three rural districts (Mbankomo, Mfou, Obala).

Study site

The Centre Region is one of Cameroon's 10 regions, located in the center of the country. Its capital is Yaoundé, which is also the country's capital. It is the country's second-largest region, with a surface area of 68,953 km², and will have a population of 5,000,000 in 2023. The Centre Region is characterized by its humid climate and a particularly rich and well-preserved environment with an average temperature of 23°C and an average rainfall of 1,747 mm. This region was chosen because it is cosmopolitan, with the majority of its inhabitants coming from all over Cameroon and even from neighboring countries such as Chad, the Central African Republic, and Nigeria.

Population

The study population consisted of healthcare professionals working in the selected health districts and parents (father and mother with children under 13 years of age) attending a health facility in one of the districts.

Inclusion criteria

This study included parents of children under the age of 13 and key health professionals involved in any of the following tasks: vaccination, consultation, and health facility management.

Exclusion criteria

Healthcare professionals not involved in prescribing and/or administering vaccines were excluded from this study.

Sample size

A total of 257 participants were recruited, including 168 parents and 89 health professionals, as follows: three health professionals per health district staff in the six health districts concerned, in particular, the key personnel involved in immunization at the district level, such as the district head, the EPI focal point, and the person in charge of the health information system; three health professionals in each of the four health facilities selected in each district (these three key personnel involved in immunization were identified by the head of the health facility and were most of the time the head of the health facility, the EPI focal point, and the doctor or nurse); and seven parents in each health facility involved.

A total of 60 interviews were conducted, 30 with parents and 30 with health professionals.

Data collection

Quantitative data were collected using two questionnaires: a first questionnaire of 60 closed questions for parents and a second questionnaire of 50 closed questions for healthcare professionals. After a pre-test and validation phase, the questionnaires were administered face-to-face. The questions covered the following themes: (a) preventive health practice and perceptions of medical care services and healthcare providers; (b) knowledge, perceptions, and attitudes towards vaccination and sources of information on vaccination; (c) knowledge of cervical cancer, HPV, and the HPV vaccine; and (d) self-reported acceptability of the HPV vaccine and its introduction into the EPI, barriers to HPV vaccination, etc.

Qualitative data collection was based on semi-structured interviews with health professionals and parents in all the health facilities involved. The main themes explored were (1) vaccination attitudes in general, (2) knowledge of cervical cancer, HPV, and the HPV vaccine, and (3) acceptability of a vaccination campaign. Using an interview guide, we favored the "conversation" format to avoid "questions and answers." The interviews were conducted in a friendly manner so as to get to know the participants and give them the opportunity to express themselves freely. A dictaphone was used to record the interviews. The interviews were transcribed in extenso.

Ethical considerations

This study obtained the authorization of the Regional Delegation of Public Health (N°0472/AP/MINSANTE/SG/DRSPC/CRERSH_19_03_ 2019) and the health authorities of the health districts concerned to be carried out. In addition, the Centre's Regional Research Ethics Committee for Human Health examined and approved this study (CE N°0463/CRERSHC/2019). In addition, an information note was given to each participant, and written informed consent was obtained from each participant before the questionnaire was administered. During the completion of the survey form, participants were identified using a code unique to each, and no individual data (surname, first name, telephone number) were recorded. The data collected was kept in a secure place and was only accessible to the researcher who conducted the study. Furthermore, verbal informed consent was required from each participant prior to the interview.

Data analysis

The quantitative data collected was entered into CSPro Version 6.2 software, and then using IBM SPSS Statistics for Windows, Version 20.0 (Released 2011; IBM Corp., Armonk, New York, United States), we carried out a simple descriptive analysis based on the data collected. For the qualitative analysis, we repeatedly read the transcribed interviews. This work enabled us to identify the significant themes emerging from the interviewees' discourse.

## Results

Quantitative results

Socio-demographic Characteristics

A total of 257 individuals were interviewed, including 168 parents and 89 healthcare professionals. The majority of parents and healthcare professionals were women (76.2% and 67.4%, respectively) (Table 1).

**Table 1:** Socio-demographic characteristics

Variable	Healthcare professionals (N=89)	Parents (N=168)
	n (%)	n (%)
Sex		
Male	29 (32.6%)	40 (23.8%)
Female	60 (67.4 %)	128 (76.2 %)
Marital status		
Married	63 (70.8%)	117 (69.6%)
Single	26 (29.2%)	49 (29.2%)
Divorced	0	2 (1.2%)
Level of education		
No education	0	2 (1.2%)
Primary school	0	27 (16.1%)
Secondary school	35 (39.3%)	73 (43.5%)
University	54 (60.7%)	66 (39.3%)
Profession (health personnel)		
Medical doctor	15 (16.9%)	-
Nurse/midwife	39 (43.8%)	-
Assistant nurse	33 (37.1%)	-
Others	2 (2.2%)	-

Knowledge and Experience of Cervical Cancer

The vast majority of healthcare professionals claim to be familiar with cervical cancer (93.3%), but only 15.7% claim to have had a screening test. A significant proportion of these professionals have experience with cervical cancer, with 1% stating that they have or have had cervical cancer and 23.6% stating that they know someone who has or has had cervical cancer. Among parents, knowledge also appears to be relatively high for a lay audience, with 54.2% claiming to know about cervical cancer. However, the screening rate is low: 7.1% of respondents. As with healthcare professionals, cervical cancer is common among the population surveyed, with 2.4% claiming to have or have had the disease and 22% claiming to know someone who has or has had it (Table 2).

**Table 2:** Knowledge and experience of cervical cancer

Variable	Parents (N=168)			Healthcare professionals (N=89)		
	Yes	No	Don't know	Yes	No	Don't know
	n (%)	n (%)	n (%)	n (%)	n (%)	n (%)
Do you know anything about cervical cancer?	91 (54.2%)	76 (45.2%)	1 (0.6%)	83 (93.3%)	6 (6.7%)	0
Have you ever been screened for cervical cancer?	12 (7.1%)	119 (70.8%)	37 (22%)	14 (15.7%)	46 (51.7%)	0
Have you ever had cervical cancer?	4 (2.4%)	101 (60.1%)	27 (16.1%)	1 (1.1%)	59 (66.3%)	0
Have you ever had genital warts?	21 (12.5%)	135 (80.4%)	12 (7.1%)	5 (5.6%)	77 (86.5%)	7 (7.9%)

Knowledge of HPV

In Table 3, in terms of knowledge about HPV and its impact on health, a lower proportion of accuracy was identified among parents for the following questions: "What is HPV?" 18.5%; "Is HPV a virus?" 23.2%; "Does carrying HPV influence cervical smear results?" 16.1%; "Is HPV a major cause of cervical cancer?" 13.7%; and "Can smoking increase the risk of cervical cancer?" 14.9%. Among healthcare professionals, on average, we can say that half of the carers questioned were aware of HPV: 60.7% said they were aware of HPV, 51.7% thought it was a virus, and 46.1% thought it could cause cervical cancer. It should be remembered that 40% of the healthcare professionals questioned are care assistants and technical and administrative staff who do not have access during their initial training to detailed knowledge, if any at all, about HPV and cervical cancer.

**Table 3:** Knowledge of HPV HPV: human papillomavirus

Variable	Parents (N=168)			Healthcare professionals (N=89)		
	Yes	No	Don't know	Yes	No	Don't know
	n (%)	n (%)	n (%)	n (%)	n (%)	n (%)
Do you know what HPV is?	31 (18.5%)	137 (81.5%)	-	54 (60.7%)	35 (39.9%)	-
Is HPV a virus?	38 (23.2%)	20 (11.9%)	109 (64.9%)	46 (51.7%)	14 (15.7%)	29 (32.6%)
Can HPV cause cervical cancer?	25 (14.9%)	17 (10.1%)	126 (75%)	41 (46.1%)	12 (13.5%)	36 (40.4%)
Does HPV carriage influence cervicovaginal smear results?	27 (16.1%)	10 (6%)	131 (78%)	38 (42.7%)	6 (6.7%)	45 (50.6%)
Is HPV a major cause of cervical cancer?	23 (13.7%)	9 (5.4%)	136 (81%)	46 (51.7%)	8 (9%)	35 (39.3%)
Can smoking increase the risk of cervical cancer?	25 (14.9%)	11 (6.5%)	132 (78%)	41 (46.1%)	9 (10.1%)	39 (43.8%)

Knowledge of HPV Vaccine

In Table 4, awareness of the HPV vaccine as a form of cervical cancer prevention was very low: 14.9% among parents and 44.9% among healthcare professionals. The timing of vaccination was less correct among parents and healthcare professionals: 14.3% and 42.7%, respectively. Around 7.7% of parents and 13.5% of healthcare professionals felt that it was not necessary to be vaccinated before sexual intercourse. A low proportion of parents and healthcare professionals (14.9% and 39.3%, respectively) answered correctly that the vaccine was harmful to health.

**Table 4:** Knowledge of the HPV vaccine HPV: human papillomavirus

Variable	Parents (N=168)			Healthcare professionals (N=89)		
	Yes	No	Don't know	Yes	No	Don't know
	n (%)	n (%)	n (%)	n (%)	n (%)	n (%)
Does the HPV vaccine prevent cervical cancer?	25 (14.9%)	12 (7.1%)	131 (78%)	40 (44.9%)	5 (5.6%)	44 (49.4%)
Should the HPV vaccine be given before the first sexual intercourse?	24 (14.3%)	6 (3.6%)	138 (82.1%)	38 (42.7%)	7 (7.9%)	44 (49.4%)
Can the HPV vaccine be given to people who have already had sex?	13 (7.7%)	15 (8.9%)	140 (83.3%)	12 (13.5%)	22 (24.7%)	55 (61.8%)
Can the HPV vaccine be harmful to health?	10 (6%)	25 (14.9%)	133 (79.2%)	4 (4.5%)	35 (39.3%)	50 (56.2%)
Can the HPV vaccine cause infection?	6 (3.6%)	27 (16.1%)	135 (80.4%)	3 (3.4%)	30 (33.7%)	56 (62.9%)
Is the HPV vaccine available in Cameroon?	7 (4.2%)	18 (10.7%)	143 (85.1%)	4 (4.5%)	31 (34.8%)	54 (60.7%)
Is the HPV vaccine included in girls' vaccination records?	7 (4.2%)	14 (8.3%)	147 (87.5%)	5 (5.6%)	34 (38.2%)	50 (56.2%)
Are three doses necessary for a complete vaccination?	17 (10.1%)	0	151 (89.9%)	23 (25.8%)	4 (4.5%)	62 (69.7%)
Does the HPV vaccine reduce the risk of developing genital warts?	21 (12.5%)	2 (1.2%)	145 (86.3%)	28 (31.5%)	1 (1.1%)	60 (67.4%)
Does the HPV vaccine reduce the risk of developing cervical cancer?	28 (16.7%)	1 (0.6%)	139 (82.7%)	39 (43.8%)	2 (2.2%)	48 (53.9%)

The proportion of incorrect answers was mixed among parents and healthcare professionals for the following questions: "Can the HPV vaccine cause HPV infection?" 3.6% and 3.4%, respectively; "Is the HPV vaccine available in Cameroon?" 10.7% and 34.8%, respectively; and "Is the HPV vaccine part of girls' vaccination records?" 8.3% and 38.2%, respectively. Parents and health professionals had a low proportion of correct responses when asked about the effectiveness of the HPV vaccine in reducing cervical cancer precursor lesions, at 16.7% and 43.8%, respectively.

Declared Acceptability of the HPV Vaccine and Its Introduction into the EPI

In Table 5, healthcare professionals were identified as having a good tendency to have affirmative answers regarding vaccine acceptance on the following questions: "Would you like to have your child or grandchild vaccinated against HPV?" 84.3% and "In your opinion, does your institution have sufficient capacity and resources to carry out HPV vaccination effectively?" 57.3%. In addition, 96.6% think that parents should be prepared for the introduction of a new vaccine. However, 42.7% of carers surveyed said they had concerns about new vaccines such as HPV.

**Table 5:** Declared acceptability of the HPV vaccine and its introduction into the EPI among healthcare professionals HPV: human papillomavirus; EPI: Expanded Program on Immunization

Variable	N=89	
	Yes	No
	n (%)	n (%)
Do you and your colleagues have any concerns about new vaccines such as the HPV vaccine?	51 (57.3%)	38 (42.7%)
Based on your professional experience, do you think parents need to be prepared for the introduction of a new vaccine?	86 (96.6%)	3 (3.4%)
Would you like to have your child or grandchild vaccinated against HPV?	75 (84.3%)	14 (15.7%)
In your opinion, does your institution have sufficient capacity and resources to carry out HPV vaccination effectively?	50 (56.2%)	39 (43.8%)
Is there a difference between public and private establishments in terms of their capacity or resources to carry out such vaccination?	51 (57.3%)	38 (42.7%)

In Table 6, parents also show a good tendency to have affirmative answers regarding the acceptance of vaccines on the following questions: "If a new vaccine is introduced in the EPI, would you like to have information about it?" 94.6%; "If a new vaccine can help prevent cervical cancer and anogenital warts: would you have your child vaccinated with this vaccine?" 80.4%; and "Would you recommend the HPV vaccine to a child, friend, or relative?" 53.3%.

**Table 6:** Parents' declared acceptability of the HPV vaccine and its introduction into the EPI HPV: human papillomavirus; TV: television; EPI: Expanded Program on Immunization

Variable	Parents (N=168)		
	Yes	No	Don't know
	n (%)	n (%)	n (%)
If a new vaccine is introduced in the EPI, would you like to know about it?	159 (94.6%)	9 (5.4%)	-
Would you be willing to have your child vaccinated to prevent cervical cancer?	139 (82.7%)	29 (17.3%)	-
If a new vaccine can help prevent cervical cancer and anogenital warts: would you have your child vaccinated with this vaccine?	135 (80.4%)	33 (19.6%)	-
If your child's doctor has recommended that he or she be vaccinated against a virus that causes cervical cancer, would you agree to have your child vaccinated, no questions asked?	70 (41.7%)	98 (58.3%)	-
Are there any issues that concern you when deciding whether or not to vaccinate your child against this virus that causes cervical cancer?	127 (75.6%)	41 (24.4%)	-
Do you think this vaccine is very important for your child?	89 (53%)	49 (29.2%)	30 (17.9%)
Would you recommend the HPV vaccine to a child, friend, or relative?	98 (53.3%)	39 (23.2%)	31 (18.5%)
How should information about the new vaccine be disseminated to promote its introduction in Cameroon?			
TV/radio	79 (47%)	-	-
Internet	44 (26.2%)	-	-
Schools	32 (19%)	-	-
Others (church, meeting, mosque)	13 (7.7%)	-	-

Source of Information on the Vaccine and Barriers to HPV Vaccination

Table 7 shows that information about the existence of an HPV vaccine remains very low among parents (83.9% never heard of it); 43.8% of healthcare professionals were informed at their training school. Parents gave a lower proportion of correct answers to the following questions: "Do you think that the HPV vaccine would stimulate the onset of sexual activity earlier?" 21.4% and "Do you think that after the HPV vaccine you should always use a condom?" 38.7%.

**Table 7:** Sources of information about the vaccine and barriers to HPV vaccination HPV: human papillomavirus; TV: television

Variable	Parents (N=168)	Healthcare professionals (N=89)
	n (%)	n (%)
Where did you hear about the HPV vaccine?		
School	4 (2.4%)	39 (43.8%)
Friends	7 (4.2%)	8 (9%)
TV/radio	7 (4.2%)	3 (3.4%)
Internet	4 (2.4%)	7 (7.9%)
Health professionals	5 (3%)	11 (12.4%)
Never heard	141 (83.9%)	21 (23.6%)
Do you think the HPV vaccine would stimulate an earlier onset of sexual activity?		
Yes	43 (25.6%)	11 (12.4%)
No	36 (21.4%)	43 (48.3%)
Don't know	89 (53%)	35 (39.3%)
Do you think you should always use a condom after the HPV vaccine?		
Yes	65 (38.7%)	43 (48.3%)
No	21 (12.5%)	27 (30.3%)
Don't know	82 (48.8%)	19 (21.3%)
Do you think you should always be screened for cervical cancer after the HPV vaccine?		
Yes	87 (51.8%)	58 (65.2%)
No	34 (20.2%)	16 (18%)
Don't know	47 (28%)	15 (16.8%)

Qualitative results

Vaccination Attitudes in General

In terms of support for vaccination, the interviews revealed a wide range of attitudes, from a radical anti-vaccination stance (one person) to pro-vaccination behavior with no declared reservations, even about new vaccines (three people). In between, attitudes were generally favorable, with a majority of children having received all the EPI vaccines and a majority of parents also wishing to have non-EPI vaccines. With regard to perceptions and experiences of children's vaccinations, we noted during our interviews that contact with health services and carers to vaccinate children is without any particular problems. For the majority, there are no undesirable effects following the vaccinations, although some report that they are not serious. Despite this, several explained that rumors are circulating about vaccines (side effects, sterility, financial interests of laboratories, etc.). These rumors have given rise to public doubts about vaccine safety. As for difficulties in accessing vaccines, several parents said they would like to have access to non-EPI vaccines, but the cost is a barrier. Some would also like to be able to catch up on missed vaccines. With regard to cases of vaccine-preventable diseases in children, during the interviews, we noted a high prevalence of vaccine-preventable diseases, since out of the 30 parents interviewed, there were three cases of measles, including one death, two cases of chickenpox, one case of poliomyelitis, one case of meningitis, and one case of tuberculosis, as illustrated by the following extracts in Table 8.

**Table 8:** Vaccination attitudes in general EPI: Expanded Program on Immunization

	Parent	Healthcare professionals
Support for vaccination	"I have 5 children. They've all been vaccinated, the vaccines you get from 0 to 9 months. They've also had the meningitis vaccine."	"No, we have no reservations."
	"Never. You can't refuse a vaccine."	
	"I have 4 of them, none of them have been vaccinated; if we have to talk about a vaccine, they have been vaccinated since they were in their mother's womb by the creator who created them, since it is God who knows his thing best, so no human hand has ever touched my children."	
Perceptions and experiences of children's vaccinations	"Everything went well (for my child's vaccination) so there were no particular problems."	"Some people don't accept the vaccine at all because they think it can make certain people sterile or cause certain diseases."
	"(for the vaccination of my children) it went very well, I had a good exchange with the nurses."	
	"I don't (I've never refused vaccines), but some people believe in conspiracy theories. They believe that vaccines are slow poisons that are injected into underdeveloped societies to regulate their population and prevent a demographic boom in certain third world countries. Others believe that when they were small, they didn't get vaccines and that didn't stop them from becoming big and strong, so their children can grow up without needing these vaccines."	
Difficulties in accessing vaccines	"There isn't much money and it's difficult to get to hospital. There's the hepatitis and meningitis vaccine that I want to have, because the disease doesn't give you milk now."	"They don't refuse, it's when they have to pay for vaccines outside the EPI that many parents refuse because they can't afford them."

Knowledge of HPV, Cervical Cancer, and Prevention Methods

With regard to knowledge and perception of HPV, with the exception of one health worker who has a perfect knowledge of this virus, the majority of people interviewed have no knowledge at all, as the following extracts show in Table 9. As for knowledge and perception of cervical cancer, knowledge of people who have had cervical cancer, and sources of information, the interviews show that overall knowledge of cervical cancer is low; but the majority have heard of it; several know people who have been affected by cervical cancer; there are many misconceptions about the causes of the disease (poor hygiene, bad sexual practices and behavior, intrauterine devices (IUDs), condoms, etc.) and about means of prevention. When it comes to knowledge and perception of the HPV vaccine, the majority have no knowledge at all, as the following extract shows (Table 9).

**Table 9:** Knowledge of HPV, cervical cancer, and prevention methods HPV: human papillomavirus; IUDs: intrauterine devices

	Parent	Healthcare professionals
Knowledge and perception of HPV	"Never heard."	"No idea"
		"Yes, the mode of transmission is sexual, and it causes cervical cancer, The best way to prevent it is to get vaccinated against HPV before having sex."
Knowledge and perception of cervical cancer	"Oh yes, it's quite common. It's caused by contraceptives like IUDs. That's a lot. Even condoms. There are also the fillings that are made there, and I haven't paid for the fillings for 20 years now. You can avoid this by practicing good hygiene, by not sticking your finger inside the condom."	"To prevent it, it's up to the hierarchy to organize by educating us and making us aware of preventive measures."
	"Yes, I think it's due to a woman's poor lifestyle. You have to adopt healthy behaviors to prevent it. Avoid dubious sexual practices."	"I don't know the causes of these cancers, not even the treatment; we can prevent them with family planning, condoms, and fidelity."
	"Cervical cancer kills. When you have it, you die. I don't really know what the causes are, but they say it's multiparity and having sex too often."	
Knowledge and perception of the HPV vaccine	"I never knew that this vaccine existed."	"I'm not familiar with this vaccine."

Acceptability of the HPV Vaccine and Vaccination Campaign

The majority of respondents were in favor of the proposed vaccination campaign, as it is important to have access to vaccines. As for their declared support for the campaign and for vaccinating their children, most are thinking of having their children vaccinated. Some explain that they would like advice from health professionals, friends, and acquaintances before making a decision, as the following extracts show. As regards the conditions for the success of this type of campaign, the majority believe that information and awareness-raising campaigns should be carried out via TV and the internet, but also through local intermediaries (teachers, local carers, opinion leaders, etc.) as illustrated by the following extracts in Table 10.

**Table 10:** Acceptability of the HPV vaccine and vaccination campaign HPV: human papillomavirus; TV: television

	Parent	Healthcare professionals
Perception towards the government's planned HPV vaccination campaign among girls aged 9-13	"I think that if the government really wants to help us, we say thank you because health is priceless. Something that will protect your health, the government really loves us so we say thank you because they've thought of us."	"This is good news, as it will make it possible to eliminate this disease."
	"A vaccination campaign is a good thing."	"It's a good thing, it will enable our young sisters to avoid cancer; it's a healthy project."
Recommending the HPV vaccine to your children and/or patients	"Some people say that it's even these vaccines that give us the disease. What's more, these vaccines are suspected of making children sterile, and that's very stressful. I'm going to take the hepatitis vaccine but I refuse to take the cancer vaccine because they say they're going to give it to a child who hasn't given birth yet. I could agree to take this vaccine with my children if they also gave it to women who had already given birth."	"Let the Cameroonian experts study the composition of the components of this vaccine and if it is proven that it is to save the population, we will adopt it."
	"Based on the information I receive from my brother, who is a doctor, or from the media, if they say the vaccine is good, that's when I'll vaccinate my daughter."	"I'm saying that if the studies are carried out and the televised debates demonstrate the reliability of the vaccine, I can recommend it to my patients, and I'll even take it myself."
		"Yes to all girls and as it's free it will help will help all girls."
Key conditions for a successful HPV vaccination campaign	"Raising awareness via TV and the internet."	"It will be beneficial to involve a number of people other than health professionals in the dissemination of this vaccine; we will certainly need to involve opinion leaders, people from the communities, and, above all, we need to build capacity because we have little information on this subject, we still have a lot of reservations about it."
	"Raising awareness."	"Inform mothers about the campaign during consultations, vaccinations, and educational talks; make representations at chiefdom level so that the chiefs can tell us which structures and associations exist in the community where we can go to talk about it."
	"It's simple: we need to involve the people who are best placed to encourage children to be vaccinated: teachers and guidance counselors… Then after the teachers, there are the parents. We need to raise their awareness."	
	"First of all, you need to show us girls who have taken this vaccine and given birth later."	

## Discussion

Vaccination attitudes in general

Overall, our study found good general support for vaccines among both healthcare professionals and the general public. However, the people interviewed stressed that there are still rumors about vaccines (financial issues, sterilization or birth control tools, fears of adverse effects in general). These concerns have been widely reported in other studies as a major obstacle to vaccination [13,14].

In fact, even if these rumors do not massively compromise adherence to vaccines (since the majority of children are well vaccinated), it seems crucial to take them into account in a vaccine communication strategy. These questions worry people and they want answers.

Knowledge of HPV, cancer, and the HPV vaccine

The qualitative data show that knowledge of HPV and the HPV vaccine is very low among the parents surveyed. This result is confirmed by the quantitative data, which show that less than 20% of those questioned had a basic knowledge of HPV and the HPV vaccine. The terse responses to questions about HPV and the vaccine given by both parents and healthcare professionals during the interviews ("No, never heard of HPV," "Never heard of it, I'm just discovering it, I didn't know it existed") underline the lack of knowledge about the virus and the vaccine that the questionnaire approach cannot fully reveal. Knowledge of HPV is considered low among respondents in several countries [15]. This finding suggests the need to reaffirm the importance of sex education programs and information about the disease and the vaccine.

With regard to cervical cancer, quantitative data show that the population is fairly familiar with this cancer (54.2% say they know about it, and over 24% have had or know someone who has). The qualitative interviews also revealed a certain awareness of this cancer. But above all, they show that there are many misconceptions (representations) in the population about this cancer and its causes: poor hygiene, deviant sexual practices, side effects of medical tools (IUD, condoms), etc. They also show that the means of prevention are poorly known (follow-up with smear tests and/or vaccination). On the other hand, our quantitative results show that access to prevention through screening remains very limited.

HPV vaccination campaign and reported acceptability of the vaccine

As regards acceptance of the HPV vaccine, the quantitative and qualitative results point in the same direction. The majority of parents and healthcare professionals are in favor of a campaign and access to this new vaccine via the EPI. However, many of them explained that they would like more information before making a decision. Around 94.6% of parents said they would like information about the vaccine. The interviews made it possible to specify the information they were looking for.

The people interviewed were primarily concerned about the safety of the vaccine. These questions appear to be all the more strategic in terms of support for the vaccine, given that the target audience for vaccination (young girls who have not had sexual intercourse) leads parents to believe rumors about the vaccine and sterility. In addition, the interviews show that, in addition to information, people are asking for testimonials in order to be convinced.: "First of all, we need to see girls who have taken this vaccine and given birth later" (parents) and "Let the Cameroonian experts study the composition of the components of this vaccine and if it is proven that it is to save the population, we will adopt it" (healthcare professionals). This behavior has already been observed among parents and healthcare professionals in other studies [16]. These results suggest that exposure to factual information about cervical cancer and the HPV vaccine is not a sufficient condition for shaping people's attitudes to HPV vaccination. Patient testimonials and the status of the people involved in raising awareness (local relays) seem to be crucial.

The information campaign should also address the issue of cervical cancer and its causes and means of prevention. In the interviews, many of the people interviewed associated cervical cancer with poor hygiene and/or deviant sexual practices. The statistical results confirm this association between HPV and deviant sexuality, with 25.6% of parents believing that the vaccine could be responsible for earlier sexual activity. To understand the value of the vaccine, it is therefore essential to provide information on the prevalence of HPV infections, the oncogenic power of these viruses, and the absence of any link between HPV infection and deviant sexual practices.

These are further arguments in favor of a major effort to communicate and inform the public before the HPV vaccine is introduced.

Limitations of the study

Our study has some limitations. Given the limited resources, our study did not include the views of other key stakeholders likely to influence the acceptability of the HPV vaccine introduction program, including religious leaders, community leaders, teachers, and adolescents.

## Conclusions

For the campaign to be successful, it is essential to understand the following points: First is the central role of information and awareness-raising about cervical cancer. The idea here is to provide people with the information they need to understand the importance of the vaccine in cervical cancer prevention. Second is the major role of information on vaccine safety: providing more general information on vaccines and undesirable effects, vaccines and commercial issues, etc. Also, specific information must be provided on the HPV vaccine and the absence of any link between infertility and sexual behavior. Third is the dissemination of information not only through the media such as TV and the internet but also through key local players (teachers, religious and community leaders, carers, and even expert patients who can talk about their illness or their experience of the vaccine).
